# Increased ischemic stroke risk in patients with Behçet’s disease: A nationwide population-based cohort study

**DOI:** 10.1371/journal.pone.0218652

**Published:** 2019-06-25

**Authors:** Ching-Ying Wu, Hsin-Su Yu, Chee-Yin Chai, Yen-Hsia Wen, Shihn-Sheng Wu, Yang-Pei Chang, Chun-Hung Richard Lin, Jui-Hsiu Tsai

**Affiliations:** 1 Department of Dermatology, Kaohsiung Municipal Ta-Tung Hospital, Kaohsiung Medical University Hospital, Kaohsiung, Taiwan; 2 Graduate Institute of Medicine, College of Medicine, Kaohsiung Medical University, Kaohsiung, Taiwan; 3 Department of Cosmetic Science, College of Human Ecology, Chang Gung University of Science and Technology, Kweishan, Taoyuan, Taiwan; 4 Department of Dermatology, Kaohsiung Medical University Hospital, Kaohsiung, Taiwan; 5 Graduate Institute of Clinical Medicine, Faculty of Medicine, Kaohsiung Medical University, Kaohsiung, Taiwan; 6 Department of Pathology, Kaohsiung Medical University Hospital, Kaohsiung Medical University, Kaohsiung, Taiwan; 7 School of Pharmacy, College of Pharmacy, Kaohsiung Medical University, Kaohsiung, Taiwan; 8 Department of Neurology, Kaohsiung Municipal Ta-Tung Hospital, Kaohsiung Medical University, Kaohsiung, Taiwan; 9 Department of Computer Science and Engineering, National Sun Yat-sen University, Kaohsiung, Taiwan; 10 Department of Psychiatry, Dalin Tzu Chi Hospital, Buddhist Tzu Chi Medical Foundation, Chia-Yi, Taiwan; Columbia University Medical Center, UNITED STATES

## Abstract

**Background:**

Behçet’s disease (BD) is a recurrent, multisystemic, inflammatory disorder that mainly affects blood vessels. Because recurrent inflammation of blood vessels in the brain plays a crucial role in the development of ischemic stroke, we hypothesized that patients with BD might have an elevated risk of ischemic stroke. This potential association has been suggested in a few case reports, but not epidemiological studies. Hence, the present study aimed to examine the relation between BD and subsequent ischemic stroke in Taiwan using a nationwide, population-based database.

**Methods:**

To establish a study cohort, the longitudinal data of 306 patients newly diagnosed with BD during 2000–2010 were extracted from the National Health Insurance Research Database, Taiwan. For comparison of ischemic stroke incidence, a control cohort of 1224 subjects without BD was established using a frequency-matched ratio of 1:4 for age, sex, and pre-existing comorbidities.

**Results:**

During the 10-year follow-up, 13 (4.2%) patients with BD and 20 (1.6%) control subjects experienced ischemic stroke. Kaplan–Meier analysis revealed the higher prevalence of ischemic stroke in the BD group (log-rank test, p = 0.001). After adjusting for comorbidities and demographic characteristics, Cox regression analysis revealed that patients with BD had a 2.77-fold risk of ischemic stroke (95% confidence interval, 1.38–5.57) compared to control subjects.

**Conclusions:**

Patients with BD have an elevated risk of ischemic stroke. Hence, BD may affect the vascular system in the brain, resulting in a stroke event.

## Introduction

Behçet’s disease (BD) is an autoimmune inflammatory disease characterized by recurrent mucosal aphthous ulcers (primarily oral but also genital) and a variety of systemic symptoms, including skin lesions as well as ocular, neurological, articular, and gastrointestinal manifestations [1, 2]. The epidemiological distribution of BD is intriguing, as it is most common in areas along the ancient Silk Road that ran from the Mediterranean Sea to Eastern Asia. The worldwide prevalence of BD is highest in Turkey and lowest in North America and Europe [3–5]. The incidence of BD in Taiwan is moderate (2.40 per 100 000 person-years), a rate that is between those of the Middle East and Europe, the Americas, and Africa [6].

Studies on the etiopathogenesis of BD currently suggest an autoimmune origin [7, 8]. Further influences thought to contribute to BD development include genetic factors, altered host–bacteria interactions, vascular endothelial activation, hypercoagulation status, aberrant immune activity, the presence of immune complexes and autoantibodies, and alterations in hematopoietic cell populations and their associated cytokines [9–11]. Clinically, BD symptoms are primarily caused by vasculitis that affects arteries and veins of various sizes. Subtle but diffusely distributed vasculitis can lead to endothelial damage; such damage can be complicated by aberrant vascular endothelial activation and hypercoagulability status in BD patients. Therefore, the likelihood of developing thrombus, a precursor to ischemic stroke, is higher in BD patients, demonstrated by the finding that BD patients often develop cerebral arterial thrombosis [12, 13]. Thus, we hypothesize that BD patients may be at elevated risk of ischemic stroke.

In Taiwan, stroke is the most prevalent cause of severe disabilities (incidence of 3.29 ‰; prevalence of 1.93%) [14, 15] and the third most prevalent cause of death [16]. Ischemic stroke is the most common stroke type, and the majority of ischemic strokes involve small vessel occlusion. Age, sex, family history of stroke, socioeconomic status, smoking, alcohol consumption, hyperlipidemia, diabetes mellitus, hypertension, obesity, and atrial fibrillation are important risk factors for stroke in the general population [14, 15, 17–25]. While, the prevalence and potential risk factors for ischemic stroke in BD patients in Taiwan remain unknown. Although multiple factors are believed to increase the likelihood of acute ischemic stroke in the general public population, the higher risk of ischemic stroke in BD patients with vasculitis warrants further assessment. To date, only few published epidemiological studies investigate the association between BD and acute ischemic stroke events [26, 27]. Therefore, the present study aimed to evaluate the risk of ischemic stroke in BD patients using a nationwide, population-based database.

## Materials and methods

### Data source and ethical consideration

Since its implementation in 1995, the National Health Insurance (NHI) program of Taiwan has provided comprehensive, unified, and universal health care services to over 99% of the Taiwanese population [28]. We used a subset of the NHI Research Data (NHIRD) that includes the claims data of one million NHI enrollees (approximately 5% of the total Taiwanese population) randomly sampled in 2000. The data cover the period from 1995 to 2010. The NHIRD contains information on patient demographics, diagnoses, medication categories and details, prescription dates, drug dosages, and treatment duration. The protocol of the present study was approved by the Institutional Review Board of Kaohsiung Medical University Hospital (KMUHIRB-EXEMPT(I)-20160003). The requirement of patient informed consent was waived because all data were extracted from the NHIRD, which is encrypted.

### Study population

The study cohort comprised patients with BD who were diagnosed according to the International Study Group criteria published in 1990 and the International Classification of Diseases, Ninth Revision, Clinical Modification (ICD-9-CM) diagnostic criteria (ICD-9-CM code 136.1) between January 1, 2000, and December 31, 2009. To increase the diagnostic validity, we specifically selected the patients who had inpatient diagnosis records with a primary or secondary diagnosis, outpatient diagnosis records with at least three consistent diagnoses, or a catastrophic illness registration card for BD. In Taiwan, BD patients can apply for a catastrophic illness registration card from the NHI Bureau, which exempts BD patients from copayments when seeking health care for BD. We defined the patient’s first visit for the diagnosis of BD as the index date. Because this study examined the risk of ischemic stroke in BD patients, those who had received a diagnosis of any type of ischemic stroke (ICD-9-CM codes 433–438) before their BD diagnosis were excluded. We also recorded the presence, before their index date, of the following potential risk factors for stroke: a past history of hypertension (ICD-9-CM codes 401–405), hyperlipidemia (272.4), diabetes mellitus (250), valvular heart disease (398.9 and 424.0–424.3), and congestive heart failure (428.xx) [15, 19, 22–25].

To establish a control cohort, control subjects were selected after frequency-matching at a ratio of 1:4 for the following characteristics: age; sex; and pre-existing comorbidities, including hypertension, hyperlipidemia, diabetes mellitus, valvular heart disease, and congestive heart failure [15, 19, 22–25].

### Ischemic stroke event measurement

The NHIRD is extensively used in Taiwan by scholars conducting pharmacoepidemiological researches. One study confirmed the validity of using the NHIRD for identifying patients who have received a principal diagnosis of ischemic stroke [29]. An ischemic stroke event was identified when any of the following criteria were met: (1) hospitalization claims; (2) four or more consecutive outpatient hospital visits, accompanied by either claims regarding the use of various types of neurological imaging techniques (magnetic resonance imaging, computed tomography, and carotid or transcranial Doppler sonography) or ischemic-stroke–related long-term prescriptions; and (3) claims for ischemic-stroke-related rehabilitation and long-term prescriptions [30]. The specificity and sensitivity of identifying ischemic stroke were 95% and 100%, respectively [31]. Related studies have reported a similar definition of ischemic stroke and have provided additional details [31, 32]. Identifying ischemic stroke by using insurance claims has been confirmed to be valid and was applied in a similar study [29]. The main outcome of the present study was the incidence of ischemic stroke after the index date. All participants were followed until either ischemic stroke after the index date, death, the end of follow-up in medical records, or the end of 2010.

### Statistical analysis

For descriptive analysis of the BD and control cohorts, continuous variables were presented as means and standard deviations, while categorical variables were reported as numbers and percentages. Intergroup differences in continuous data were determined using Student’s *t*-test. The chi-square or Fisher's exact test was employed for evaluating intergroup differences in categorical or proportional variables, as appropriate. Ischemic-stroke–free survival was analyzed using the Kaplan–Meier method, with significance calculated using the log-rank test. Cox proportional hazard regression was used for multiple regression analysis. Statistical significance was defined as two-sided p < 0.05. All data were analyzed using IBM SPSS version 24.0 (IBM Corp, Armonk, NY, USA).

## Results

### Demographic characteristics of the study population

The present study included 306 BD patients and 1224 control subjects without BD. Table 1 summaries the demographic characteristics and selected comorbidities for these two groups. The mean age of the BD patients was 40.31 ± 14.86 years, and 57.84% of the BD patients were women. The comorbidity prevalence (e.g., cardiovascular diseases) did not differ significantly between the groups, as expected. The median follow-up periods of the BD and control groups were 3.48 years (range, 1.01–5.95) and 3.57 years (range, 0.62–6.51), respectively. Of the total 1530 participants, 33 (2.15%) experienced stroke during the follow-up period (Table 1), with 13 (4.25%) in the BD cohort and 20 (1.63%) in the control cohort.

**Table 1:** Comparison of baseline characteristics between subjects with and without BD.

	Behçet’s disease (n = 306)		Comparisons (n = 1224)		p
**Age, years**	40.31 ± 14.86		40.31 ± 14.84		1.000
**Male**	129	42.16%	516	42.16%	1.000
**Comorbidities**					
Hypertension	72	23.53%	288	23.53%	1.000
Diabetes mellitus	34	11.11%	136	11.11%	1.000
Valvular heart disease	14	4.58%	56	4.58%	1.000
Congestive heart failure	4	1.31%	16	1.31%	1.000
Hyperlipidemia	34	11.11%	136	11.11%	1.000
**Ischemic stroke**					
Occurrence	13	4.25%	20	1.63%	0.005
Duration, days	1269.00 ± 902.04		1302.75 ± 1074.65		0.506

BD, Behçet’s Disease.

### Risk factors for ischemic stroke in patients with BD

**Fig 1:**
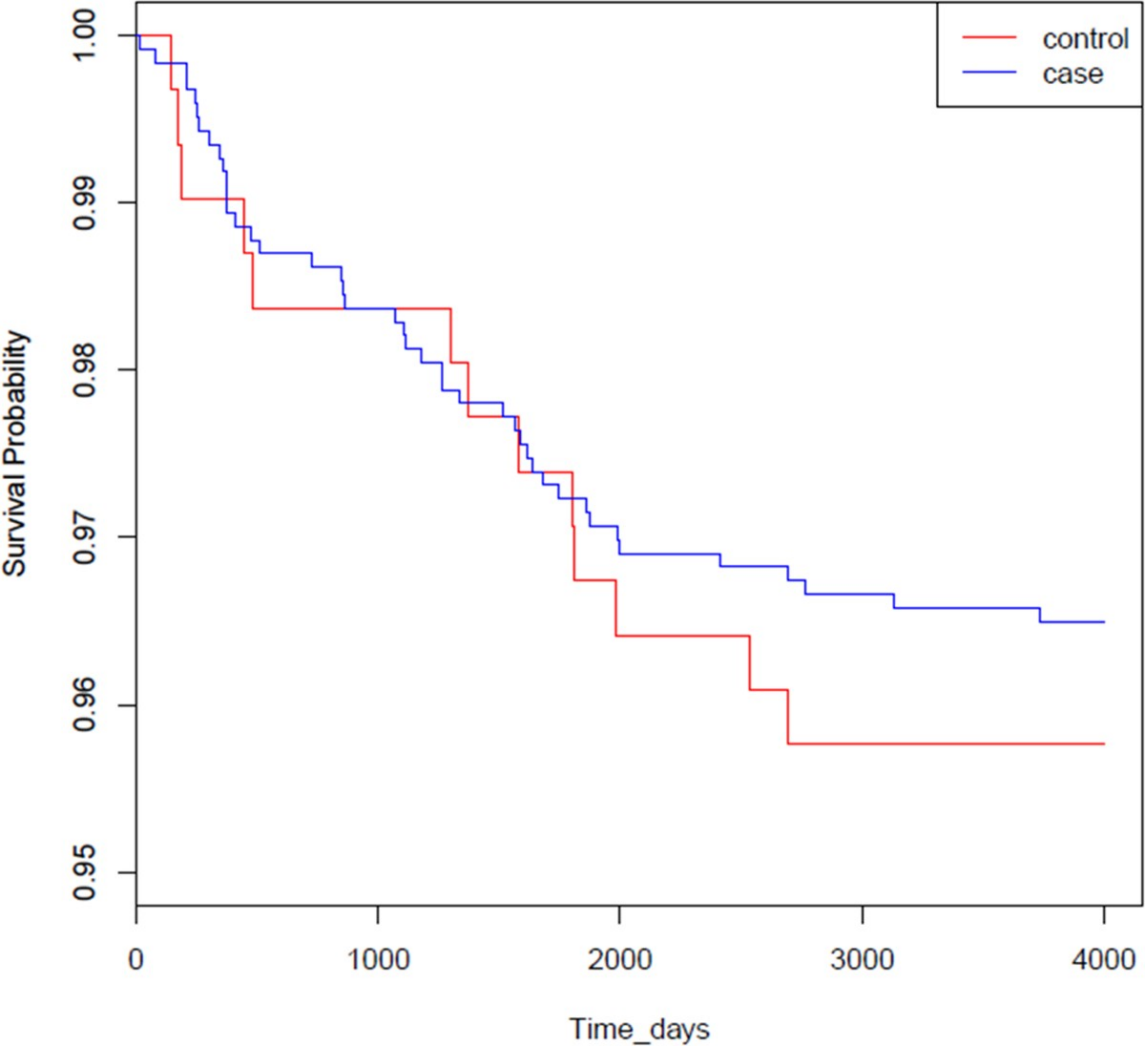
Kaplan–Meier curves of freedom from ischemic stroke between patients with Behçet’s Disease (BD) and the comparisons without. Group 1, patients with BD; group 2, control subjects without BD. The difference between the two curves is statistically significant (log-rank test, p < 0.01).

Kaplan–Meier and log-rank analyses indicated a significantly higher prevalence of ischemic stroke in the BD group compared with the control group (p, 0.004) (Fig 1), indicating that BD patients had an increased ischemic stroke risk over the study period. Univariate Cox regression analysis revealed that compared with the control group, the BD group had a crude hazard ratio (HR) for ischemic stroke of 2.690 (95% confidence interval [CI], 1.338–5.409; p, 0.005) during the follow-up period. After adjustment for age, sex, and pre-existing comorbidities, multivariate Cox regression analysis revealed that the stroke risk during the follow-up period was 2.768 times higher (95% CI, 1.376–5.570; p, 0.004) in BD patients than control subjects (Table 2), suggesting that BD patients have an increased risk of having an ischemic stroke event. Univariate analysis also revealed an increased ischemic stroke risk in BD patients who were older (crude HR, 1.057; 95% CI, 1.035–1.080) and in those with diabetes mellitus (crude HR, 4.249; 95% CI, 2.084–8.664), hypertension (crude HR, 6.980; 95% CI, 3.319–14.682), or previous congestive heart failure (crude HR, 8.859; 95% CI, 3.368–23.304) (Table 2). Furthermore, multivariate analysis showed that after adjustment for potential confounders, older age (adjusted HR, 1.030; 95% CI, 1.003–1.058; p, 0.028) and hypertension (adjusted HR, 3.672; 95% CI, 1.474–9.148; p, 0.005) were independent risk factors for ischemic stroke in BD patients (Table 2).

**Table 2:** Independent predictors of ischemic stroke in patients with BD.

Variables	Crude HR (95% CI)	p	Adjusted HR^a^ (95% CI)	p
**Age**	1.057 (1.035–1.080)	< 0.001	1.030 (1.003–1.058)	0.028
**Male**	1.021 (0.512–2.037)	0.952	0.841 (0.408–1.736)	0.640
**Hypertension**	6.980 (3.319–14.682)	< 0.001	3.672 (1.474–9.148)	0.005
**Diabetes mellitus**	4.249 (2.084–8.664)	< 0.001	1.862 (0.873–3.975)	0.108
**Valvular heart disease**	1.077 (0.257–4.509)	0.919	0.511 (0.112–2.322)	0.385
**Congestive heart failure**	8.859 (3.368–23.304)	< 0.001	2.476 (0.884–6.993)	0.084
**Hyperlipidemia**	2.719 (1.262–5.858)	0.011	0.898 (0.387–2.084)	0.802
**Behçet’s disease**	2.690 (1.338–5.409)	0.005	2.768 (1.376–5.570)	0.004

BD, Behçet’s disease; HR, Hazards ratio; CI, Confidence interval

^a^After adjustment for age, male sex, hypertension, diabetes mellitus, valvular heart disease, congestive heart failure, hyperlipidemia, and Behçet’s disease

## Discussion

Although the neurological deficits associated with BD are well recognized, the association between BD and ischemic stroke has been rarely explored [26, 33–36]. To the best of our knowledge, this study is the first nationwide population-based investigation of potential ischemic stroke risk factors in BD patients in Asia. The results revealed a significantly increased risk of ischemic stroke for BD patients compared to control subjects without BD. Moreover, old age and hypertension are independent risk factors for ischemic stroke in BD patients.

BD is a rare, chronic autoimmune disorder with a variety of etiologies [37]. The main etiologies include genetic factors; altered host responses to microbes, hematopoietic cells, and cytokines; autoantibody and immune complex formation; and altered vascular endothelial activation and hypercoagulability [9, 10]. The manifestations of BD are believed to be caused by vasculitis that results in damage to blood vessels throughout the body [38]. The pathogenesis of stroke in BD patients may be multifactorial, which remains to be a topic of debate and the focus of research. Although BD manifests as recurrent aphthous ulcers, uveitis, and genital ulcers, BD can be devastating and even fatal because of vascular aneurysm rupture or severe neurological complications [39–41].

In the present study, we observed a significantly increased risk of ischemic stroke among BD patients. The vasculitis–BD relationship may underlie this increased risk of acute ischemic stroke. Decreased endothelium-dependent flow-mediated dilatation has been observed in BD patients [42, 43]. In addition, BD patients exhibited increased intima-media thickness and decreased arterial distensibility compared with controls [44]. These factors all contribute to a tendency toward vascular stenosis in BD patients. Among stroke subtypes, ischemic stroke accounts for nearly 80% of stroke events [16]. Ischemic stroke is caused by decreased or completely blocked blood flow [45]; such reduction in the blood supply may be caused by severe stenosis, reduced systemic perfusion, or blood vessel occlusion. The primary causes of ischemia include thrombosis, embolization, and lacunar infarction resulting from small vessel disease. Therefore, thrombosis development is an important risk factor for ischemic stroke in BD patients.

Several BD-associated factors may contribute to thrombosis development to some degree in BD patients [28, 42]. Endothelial activation in the lumen of affected vessels may result in vascular inflammation and thrombosis in BD [46, 47]. Many BD patients appeared to be in a generalized hypercoagulable state, based on evidence of increased thrombin formation and decreased fibrinolysis [48, 49]. Arteritis that involves the vascular system in the brain parenchyma may lead to ischemic stroke, aneurysmal dilatation, dissection, and subarachnoid hemorrhage. In the present study, we observed a 2.77-fold higher ischemic stroke risk in BD patients.

In the present study, older age and hypertension were found to be independent factors contributing to ischemic stroke in BD patients. In addition, BD patients with older age and hypertension had a higher risk of stroke. The findings of this study show that BD patients had a considerably higher ischemic stroke risk than expected; therefore, the hazardous effects of vascular damage and hypercoagulative status on cerebral vessels should not be overlooked. A proactive and prompt preventive plan should be employed for BD patients, especially for those with concomitant risk factors for ischemic stroke. Our statistical analyses using information in a medical database do not provide sufficient information regarding the association between BD and stroke, especially regarding the mechanism of pathogenesis. However, our data show that BD patients have a significantly increased risk of ischemic stroke. Further research is necessary to confirm our findings and explore the mechanism underlying the association between BD and stroke.

### Strength and limitation in the study

The main strength of this research is its data source, a nationwide population-based database that represents the general population of Taiwan. Most events could be traced and patients followed, and referral bias was kept to a minimum because Taiwan’s NHI program is a single-payer, mandatory program with low copayments and services that cover more than 99% of the Taiwanese population. Furthermore, ischemic stroke and comorbidity diagnoses were reliable; claims on the NHI are scrutinized and validated by medical reimbursement specialists and are subject to peer review.

Nevertheless, the present study also had some limitations. Firstly, the NHIRD does not contain detailed biochemical data, body weight or body mass index details, information indicating the clinical severity of diseases, family history, or lifestyle factors, each of which may be a risk factor or comorbidity for ischemic stroke. For example, the *HLA-B51/B5* gene is recognized as a risk factor that substantially contributes to BD, but this information is not included in the NHIRD. In addition, the data regarding lifestyle-related risk factors for ischemic stroke such as dietary habits, cigarette smoking, alcohol consumption, and physical activity were unavailable. Second, this study had a retrospective design, and such studies tend to be more susceptible to bias than are those with a prospective design. However, this study avoided two possible major biases-related sampling problems by selecting patients randomly from a population-wide database and by avoiding recall problems via examining national health care records. Finally, as with the compilation of data in any administrative database, coding errors and undercoding are possible.

### Conclusions

BD Patients in Taiwan are at increased risk of ischemic stroke. More aggressive primary and secondary prevention strategies are needed for this patient population. Further investigation is necessary to determine whether treating conditions that are stroke risk factors in these patients can decrease the likelihood of ischemic stroke.
